# Supplementary figures and images for: Repression of ctrA and chpT by a transcriptional regulator of the Xre family that is expressed by RpoN3 and its cognate activator protein in Cereibacter sphaeroides
Source: PLoS One. 2025 Apr 15;20(4):e0321186. doi: 10.1371/journal.pone.0321186 (PMC11999139; doi:10.1371/journal.pone.0321186)

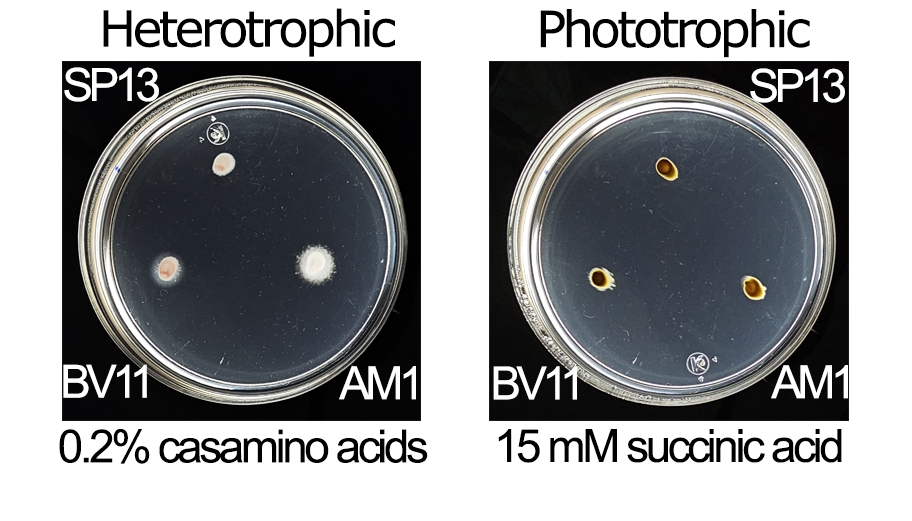

Supplement: S1 Fig — Soft-agar plates containing minimal medium containing the indicated carbon source were inoculated with the indicated strains and incubated either under photoheterotrophic or heterotrophic conditions for 48 h. (TIF) [file pone.0321186.s001.tif]

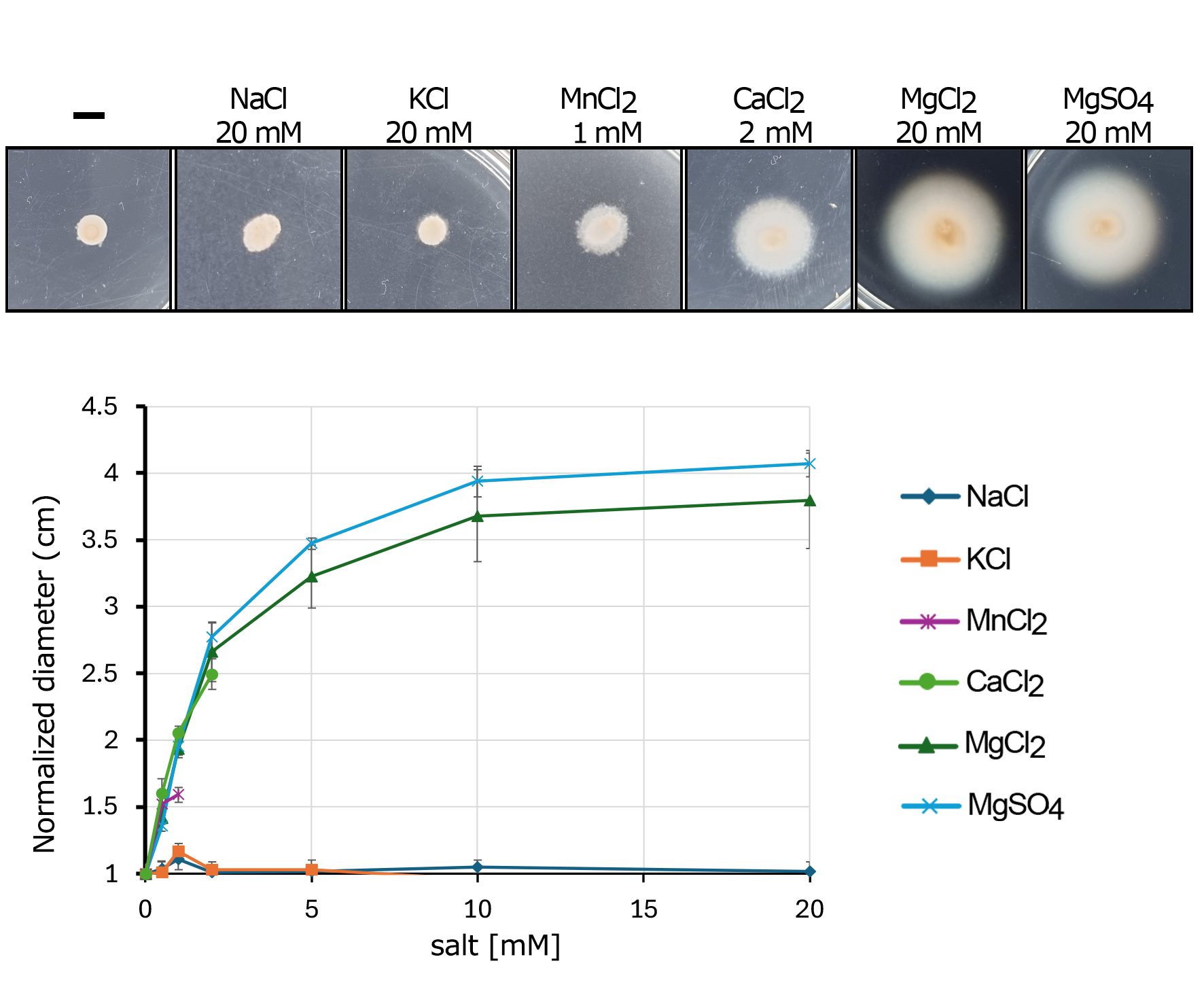

Supplement: S2 Fig — Soft-agar plates containing minimal medium with 15 mM succinic acid as the carbon source were supplemented with different salts at different concentrations. After inoculation, the plates were incubated under heterotrophic conditions for 48 h. At the top of the figure, the swimming ring formed at the highest concentration of the tested salt is shown. Below, the normalized values of the swimming ring for each salt concentration are shown. Normalization was done by subtracting the measurements taken in the absence of salt. The values determined in the absence of salt were used as the reference values. Measures represent the average of three experiments, each with four replicate swimming plates. The standard deviation is indicated. (TIF) [file pone.0321186.s002.tif]

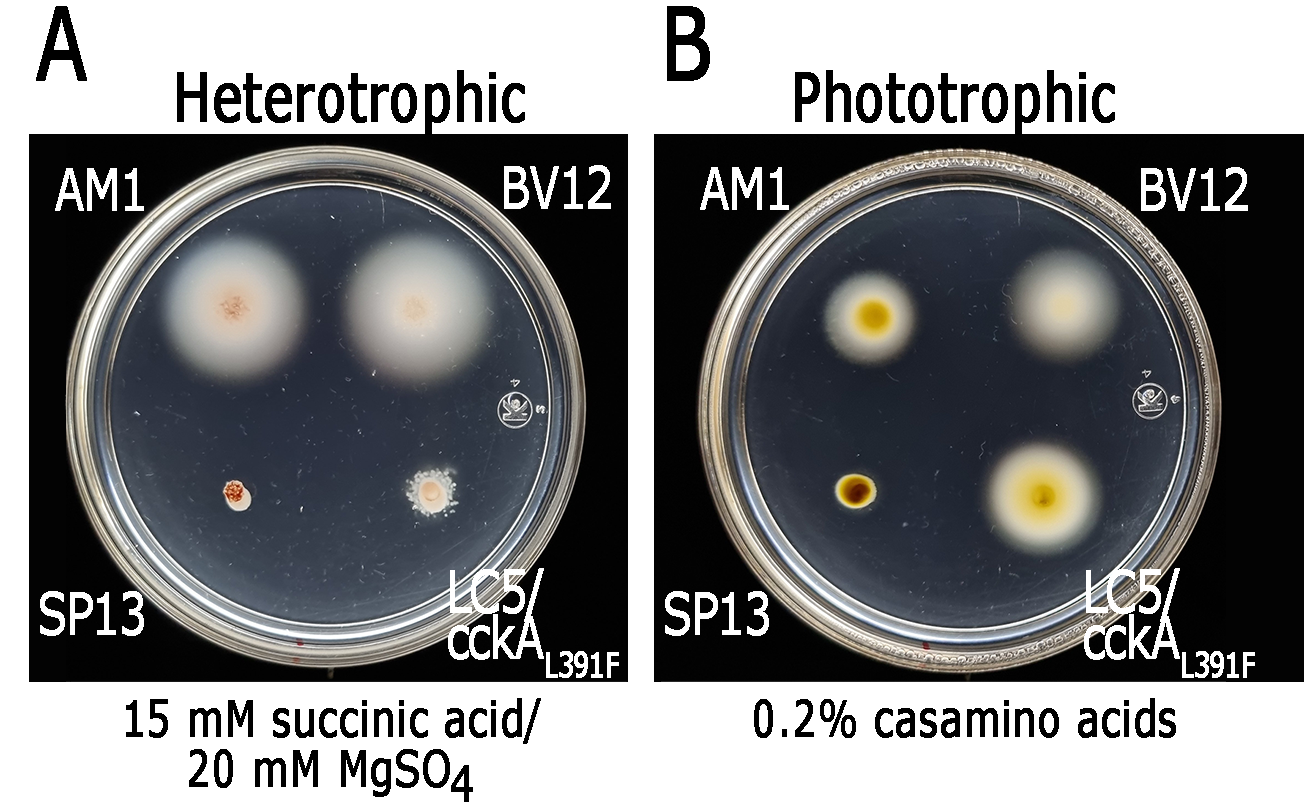

Supplement: S3 Fig — Soft-agar plates containing minimal media with the indicated carbon source were inoculated with the indicated strains and incubated either under heterotrophic or photoheterotrophic conditions for 48 h. (TIF) [file pone.0321186.s003.tif]

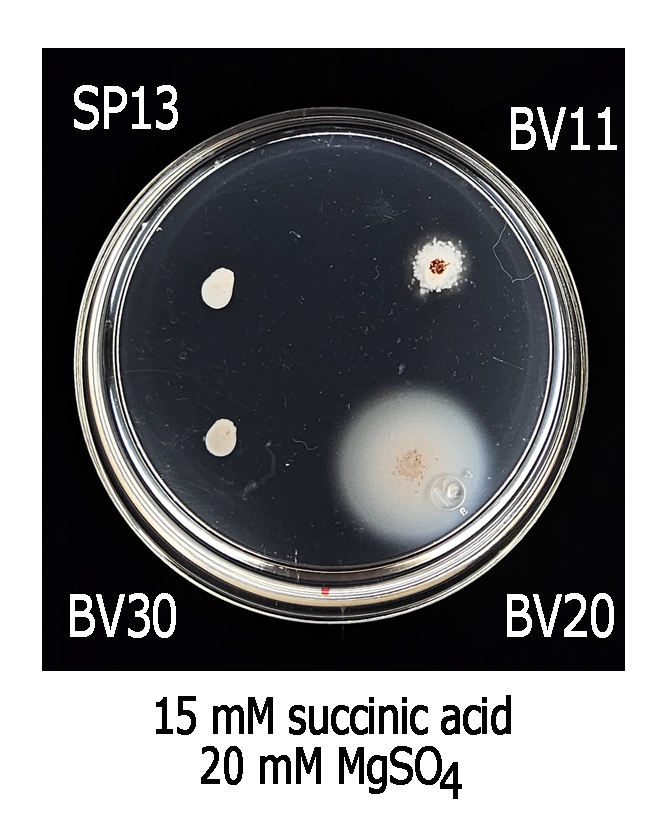

Supplement: S4 Fig — Soft-agar plates containing minimal media with 15 mM succinic acid/20 mM MgSO4 were inoculated with the indicated strains and incubated heterotrophically for 48 h. (TIF) [file pone.0321186.s004.tif]

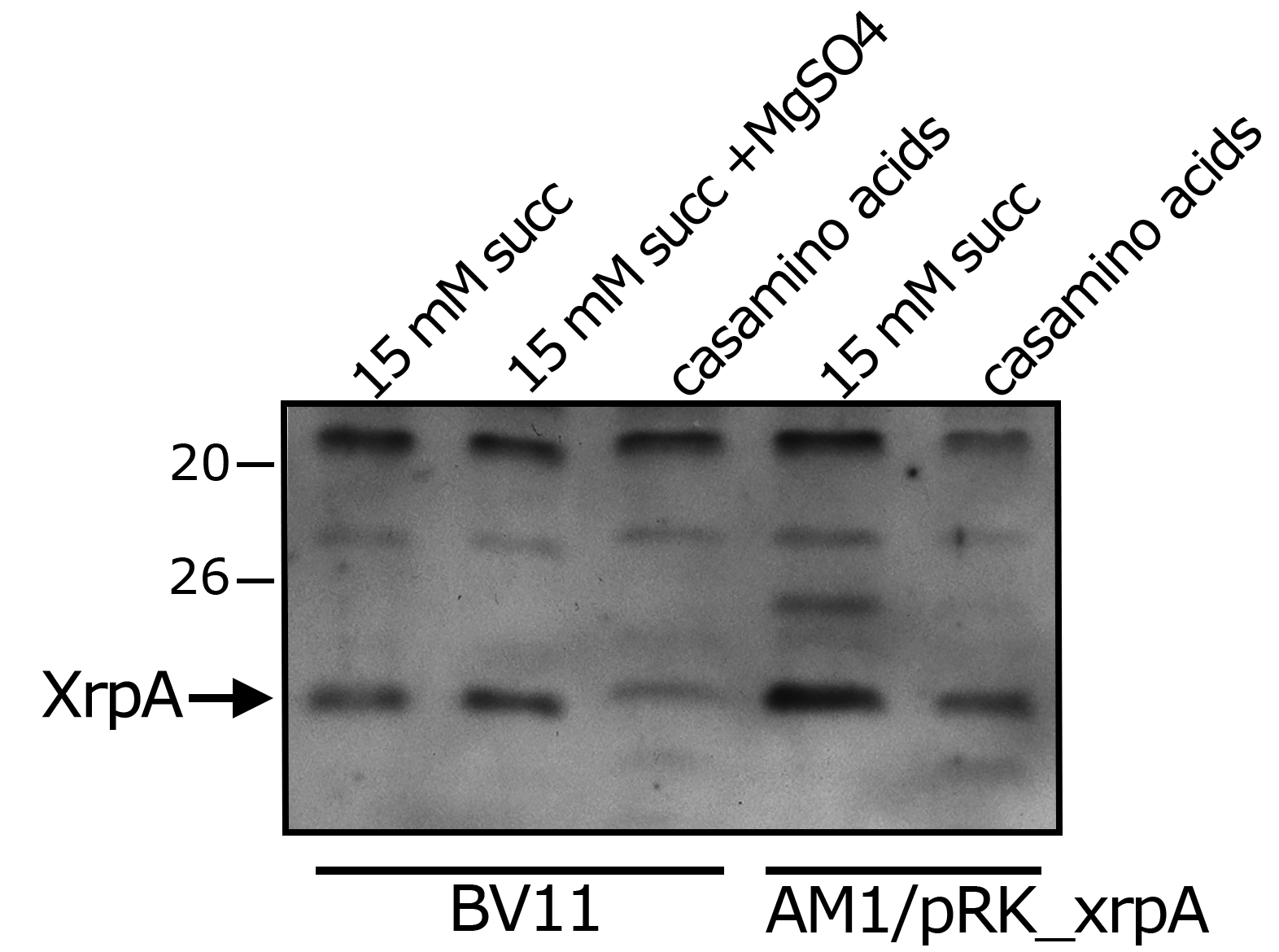

Supplement: S5 Fig — Cells were grown heterotrophically in Sistrom’s minimal medium containing 15 mM succinic acid, 15 mM succinic acid/20 mM MgSO4, or photoheterotrophically in 0.2% casamino acids. (TIF) [file pone.0321186.s005.tif]

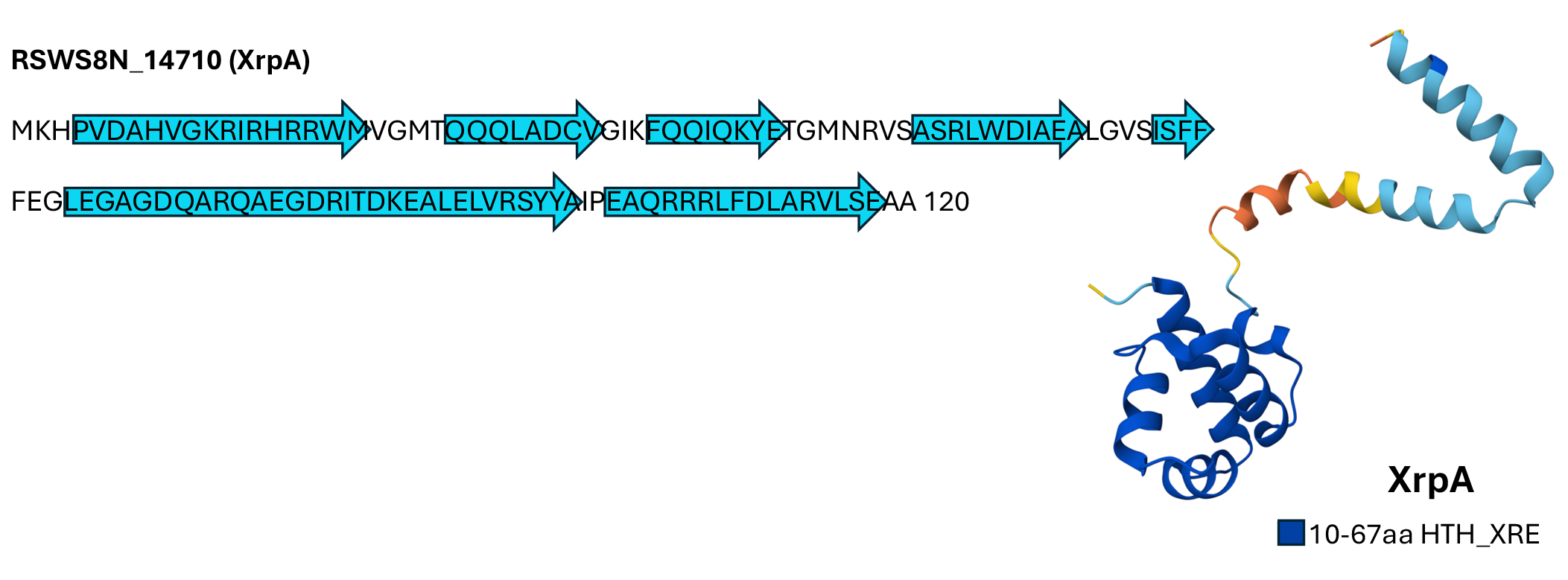

Supplement: S6 Fig — The secondary structure prediction of XrpA was obtained using Psipred [39]. Blue arrows represent α-helixes. At the left, the ribbon view of the XrpA structure predicted by AlphaFold is shown (AF-Q3J594-F1-v4 for the identical protein Q3J594/RSP_1892 from C. sphaeroides 2.4.1) [40, 41]. (TIF) [file pone.0321186.s006.tif]

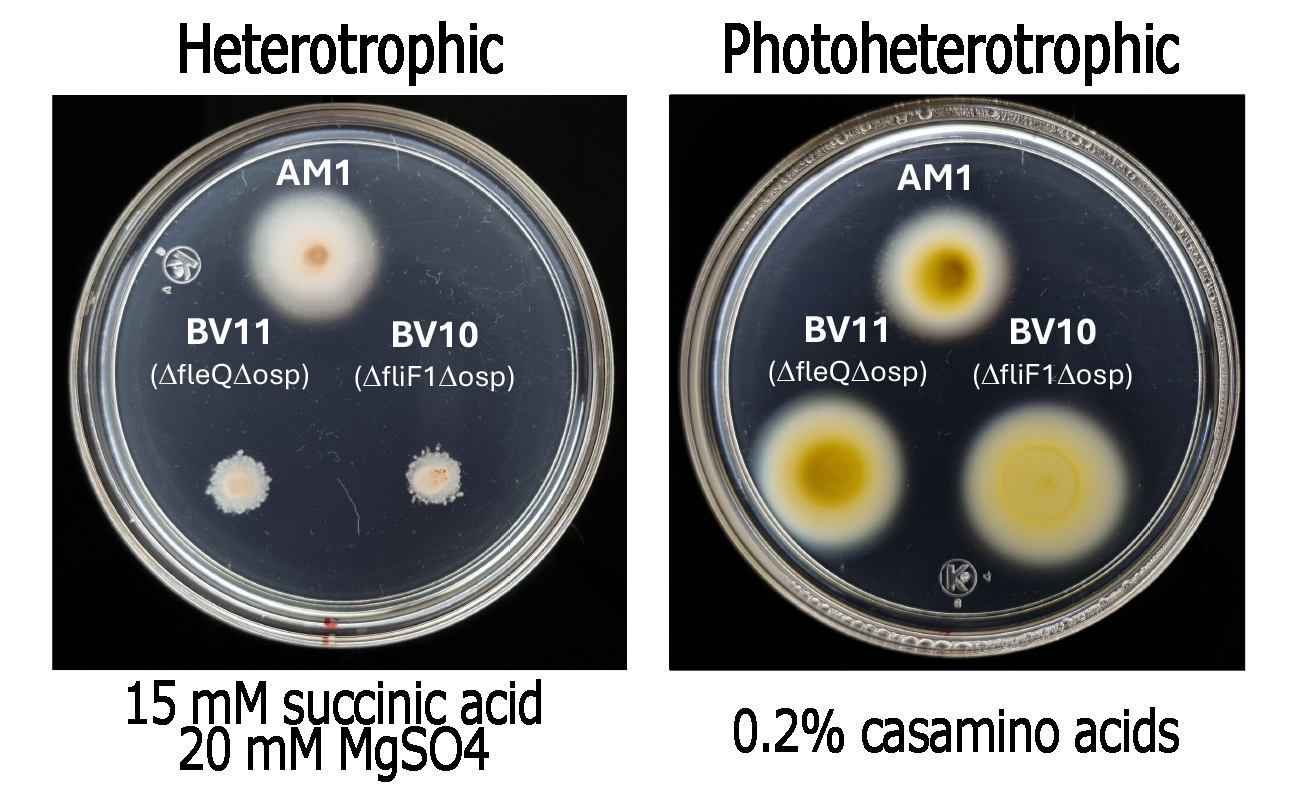

Supplement: S7 Fig — Soft-agar plates containing minimal media with the indicated carbon source were inoculated with the indicated strains and incubated under heterotrophic or photoheterotrophic conditions for 48 h. (TIF) [file pone.0321186.s007.tif]

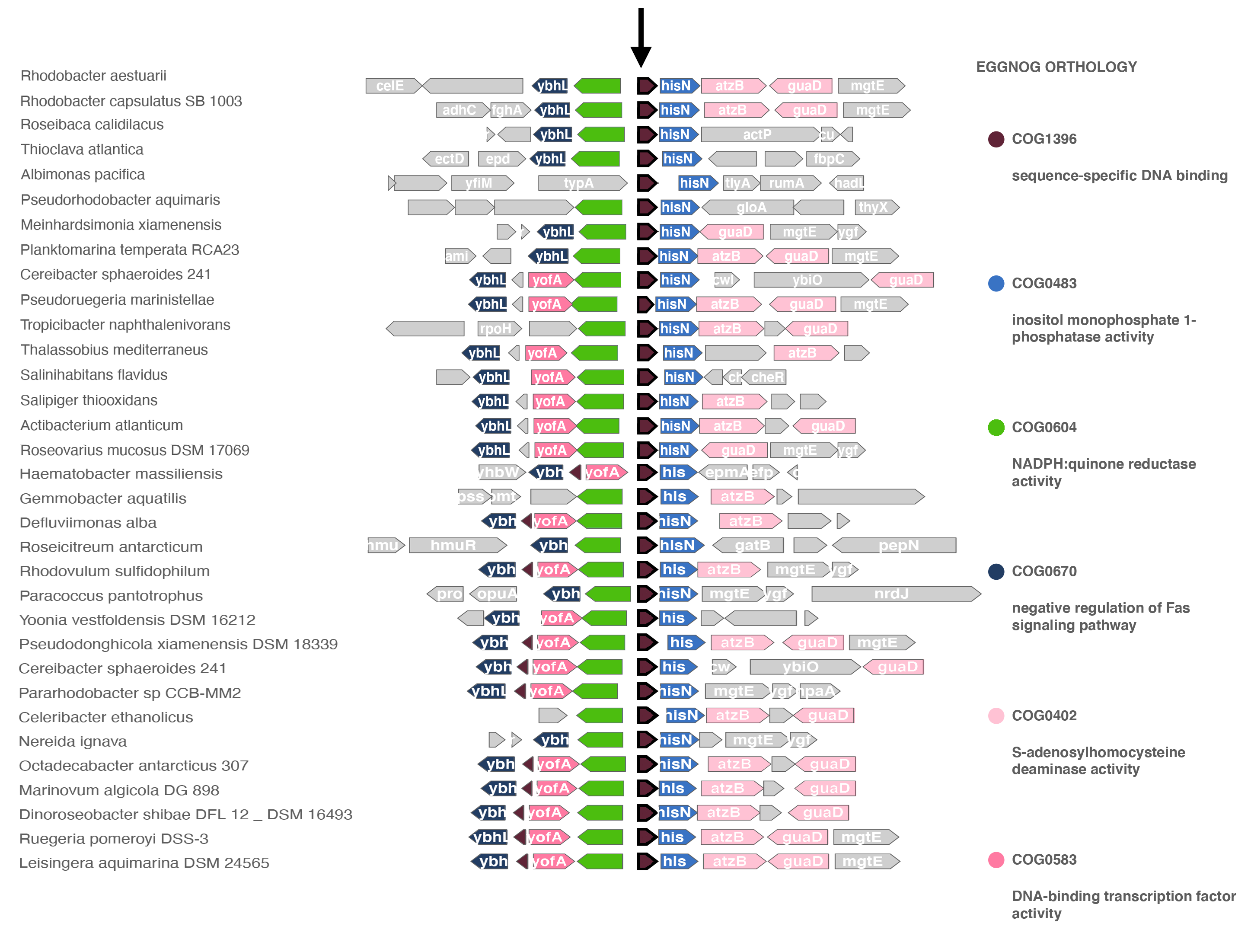

Supplement: S8 Fig — (TIF) [file pone.0321186.s008.tif]

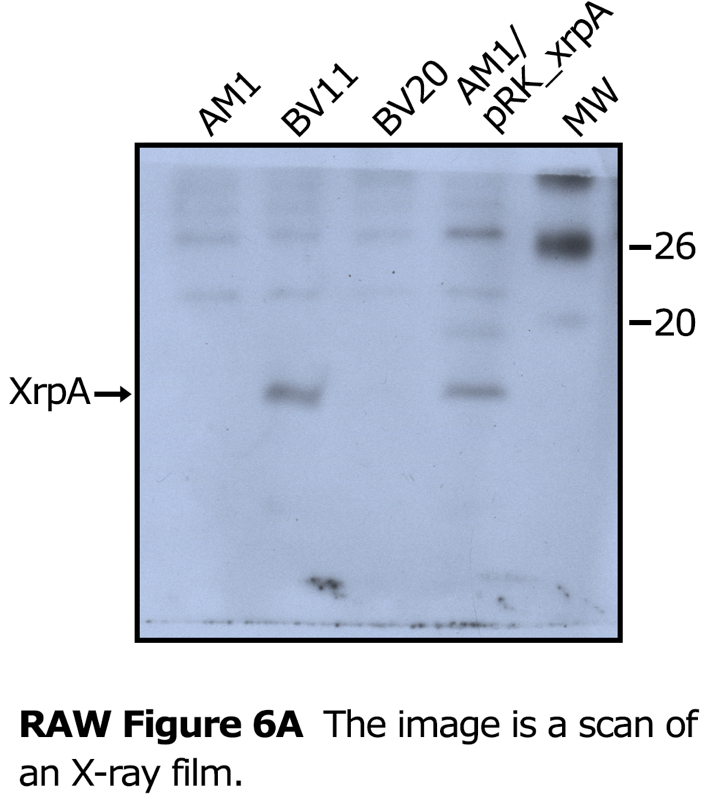


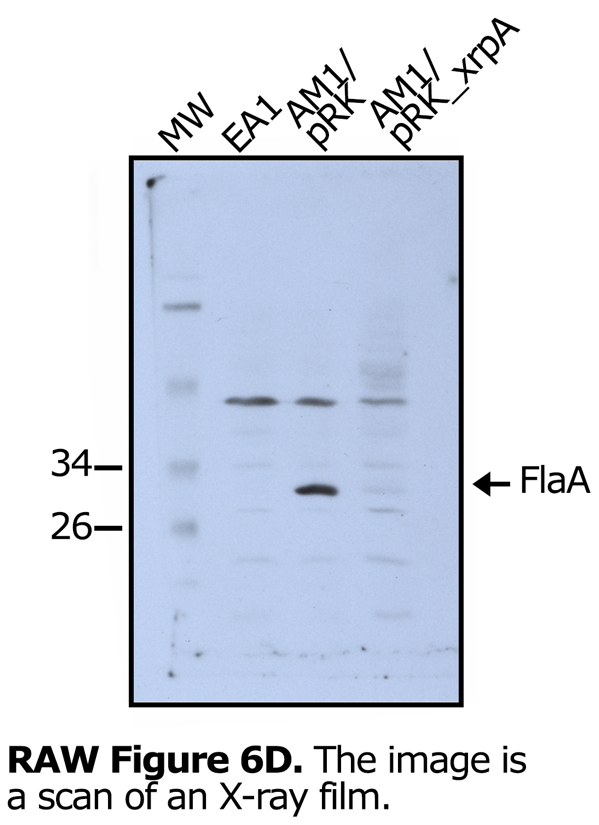


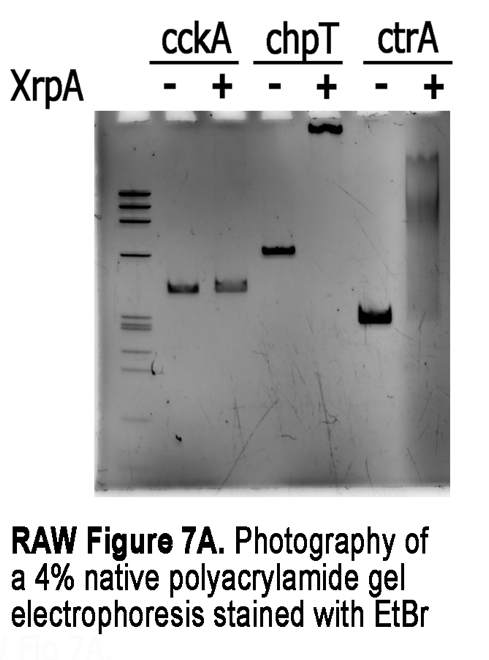


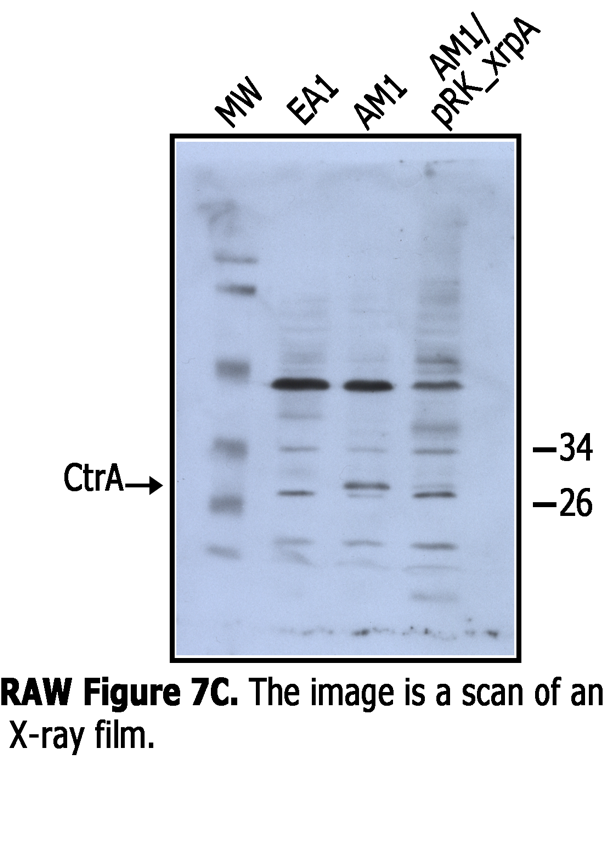


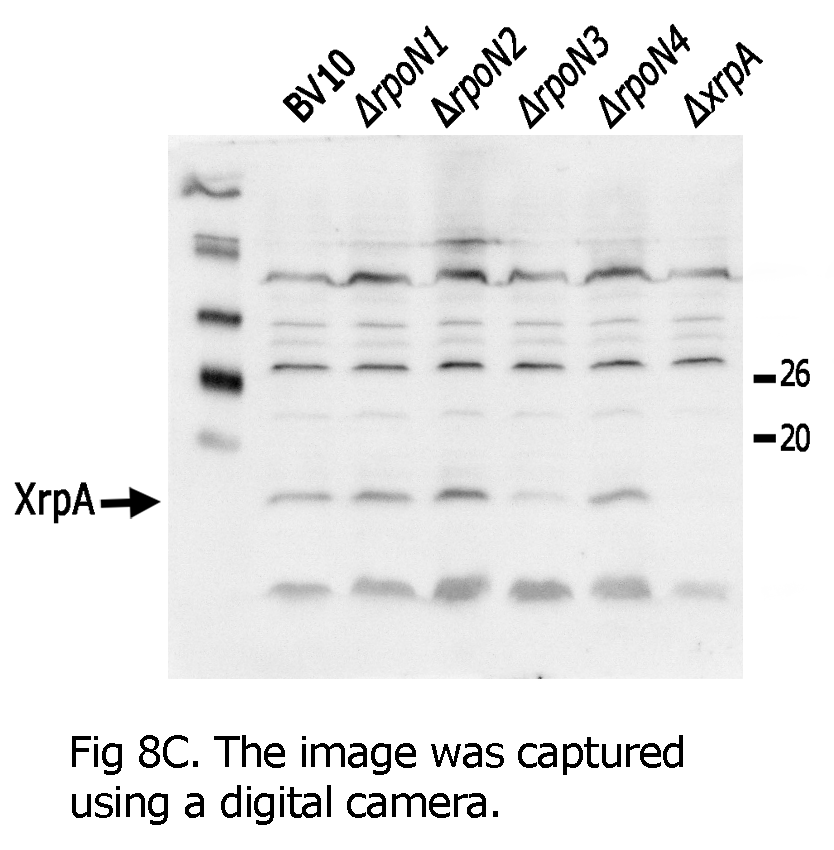


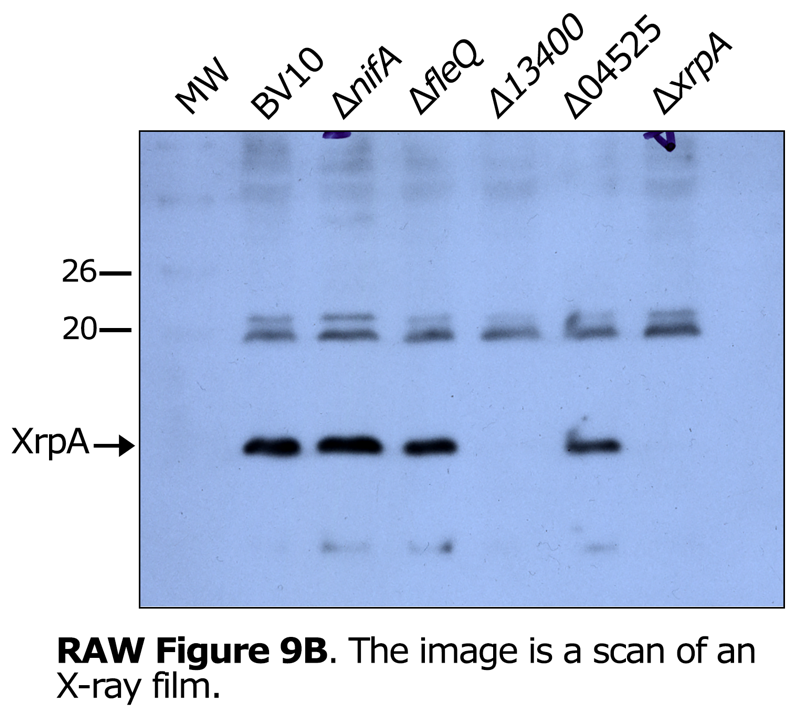


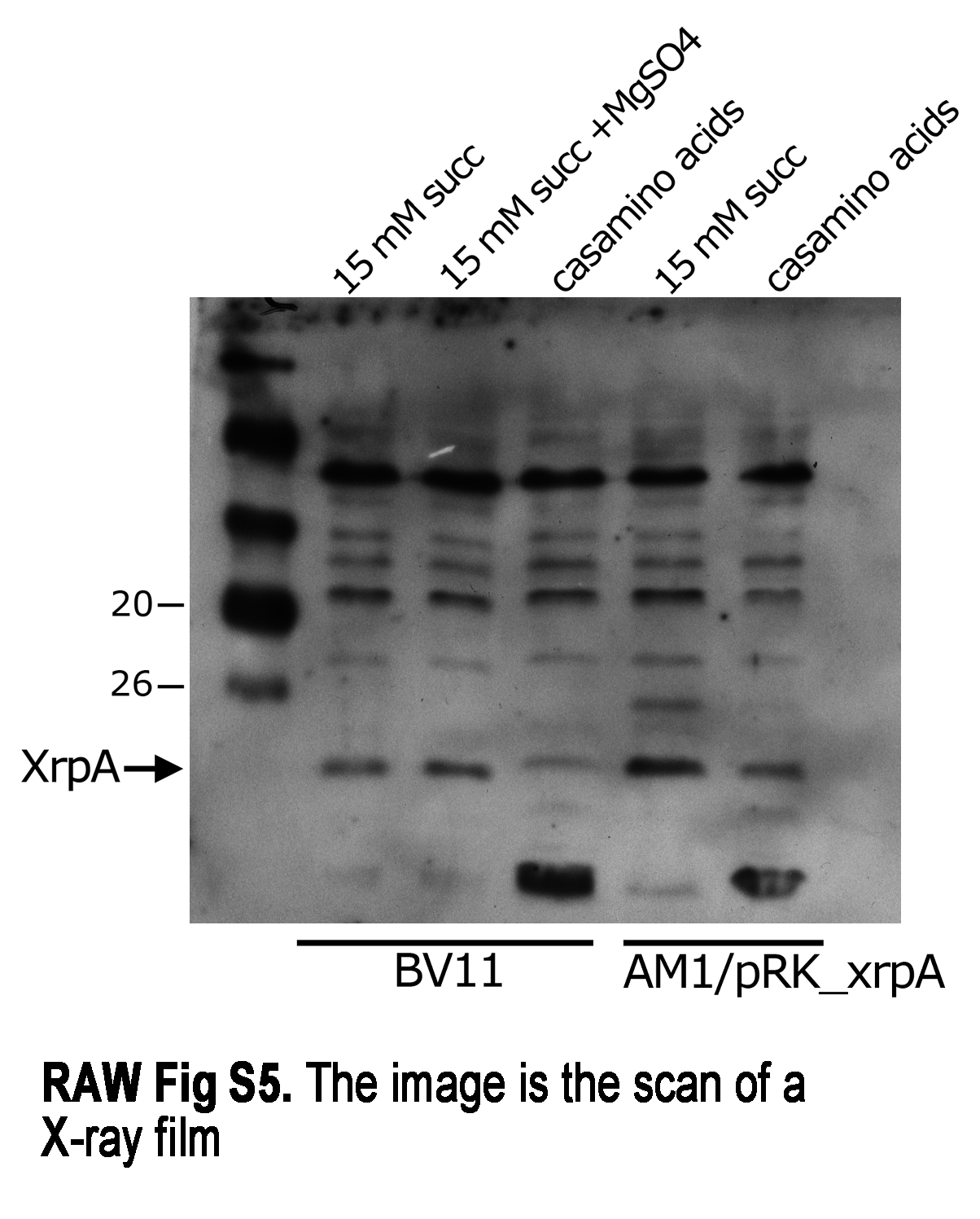

Supplement: S1 Raw Images — (DOCX) [file pone.0321186.s011.docx]
