# Supplementary material for: Repression of ctrA and chpT by a transcriptional regulator of the Xre family that is expressed by RpoN3 and its cognate activator protein in Cereibacter sphaeroides
Source: PLoS One. 2025 Apr 15;20(4):e0321186. doi: 10.1371/journal.pone.0321186 (PMC11999139; doi:10.1371/journal.pone.0321186)
Supplement: S1 Table — (DOCX) [file pone.0321186.s009.docx]

Table S1 Oligonucleotides used in this work

| Name | Sequence |
| --- | --- |
| FW xre EcoRI | GCGAATTCATGAAACATCCCGTCGACG |
| Rv Xre HindIII | GCAAGCTTCTAGGCGGCTTCGGAGAGCAC |
| Fw Xre NdeI | GCCATATGAAACATCCCGTCGACG |
| FW petMet EcoRI | GCGAATTCACCATGGGCAGCAGCCATCATC |
| Fw XREcro xba | GCTCTAGAAGCCGAAGGCCGCGACGCGCG |
| Rv XREcre sac | GCGAGCTCCGCCGCCGCGCGAGGATGGCG |
| AAD1Bam | GCGGATCCCCTGAAGCCAGGGCAGATCCG |
| AAD2Bam | GCGGATCCTCATGATATATCTCCCAATTTG |
| Fw Xre XbaI | GCTCTAGAGGGGTTTTGGCACACAATATG |
| uidCOOHXba | GCTCTAGATCATTGTTTGCCTCCCTGCTG |
| ACT159 2 | GCTCTAGACCCTTCTGCGGTTGCGCGACCAGC |
| ACT159 1 | GCTCTAGAGACGGATGGACCTTCGATCGCTCC |
| Kanfw1 Eco | GCGAATTCGGGAAAGCCACGTTGTGTCTC |
| KanrevEco | GCGAATTCGCTGAGGTCTGCCTCGTGAAG |
