# Supplementary material for: Repression of ctrA and chpT by a transcriptional regulator of the Xre family that is expressed by RpoN3 and its cognate activator protein in Cereibacter sphaeroides
Source: PLoS One. 2025 Apr 15;20(4):e0321186. doi: 10.1371/journal.pone.0321186 (PMC11999139; doi:10.1371/journal.pone.0321186)
Supplement: Attachment — Submitted filename: Response to Reviewers.docx [file pone.0321186.s013.docx]

**RESPONSE TO REVIEWERS**

*Reviewer #1: …..I suggest several modifications to the manuscript as follows.

(Figure 2) Seeing the photos under the photoheterotrophic condition, I cannot understand the difference in the expansion of swimming rings between AM1 and SP13; both strains’ rings seem to be expanded similarly by MgSO_4_. How were the ring diameters measured?
In addition, as in the other figures, please add indications of each panel by A, B, C, and D.*

(Figure 2) Swimming plates are inoculated with a 2 µl drop of each strain, if the drop slides on the surface of the culture medium, we observe a lopsided denser growth zone and if the strain can swim a less dense swimming ring appears. In Fig 2C in the presence of MgSO4, SP13 only shows the lopsided growth, while the AM1 strain exhibits the lopsided growth and the swimming ring. When this occurs, we measure the shortest diameter of the ring. This information was included in Material and Methods (lines 160-163).

As suggested, we have added indications of each panel by A, B, C and D.

*(lines 280-283) The authors state “Conversely, in plates incubated under the photoheterotrophic condition, the inclusion of MgSO₄ only slightly promoted the swimming of BOTH strains ( ~2.5-fold) (Fig. 2).” The right panels of Fig 2 show the swimming diameter of BV11under photoheterotrophic condition is larger than that under heterotrophic condition. It would be better to revise the sentence to “Conversely, in plates incubated under photoheterotrophic conditions, the inclusion of MgSO₄ only slightly promoted the swimming of AM1.” Or, please explain the enhancement of BV11 motility under the photoheterotrophic condition.*

The purpose of Figure 2A is to explain why heterotrophic growth conditions utilizing 15 mM succinic acid as a carbon source were chosen for the subsequent experiments in this work. As it can be seen, the difference in the response of the AM1 and BV11 strains to the presence of MgSO_4_ is stronger under this condition, making it more suitable to study the effect of XrpA. We have slightly modified the original text to clarify this point (lines 290-293).

*(Figure 3) It is difficult to see the scale bars in the EM photos. Please use a larger font size.*

(Figure 3) As suggested, we have increased the font size and the scale bar.

(lines 389-390) The authors state “the OVEREXPRESSION of XrpA leads to the inhibition of Fla2-dependent motility even under these conditions, in which BV11 (xrpA+) is able to swim.” Fig. 6A shows that the expression levels of XrpA from BV11 and AM1/pRK_xrpA appear to be comparable. What is the rationale for the overexpression of XrpA in AM1/pRK_xrpA?

We have modified the text to indicate that XrpA is expressed in an unregulated manner from the pRK_xrpA plasmid (not overexpressed), and this significantly reduces the swimming ability of AM1/pRK_xrpA when grown in casamino acids (line 399). To further support this idea, we have included a supplementary figure (Fig. S5) to show the differences in XrpA levels under different growth conditions for the BV11 and AM1/pRK_xrpA strains (lines 401-405).

*(Figure 12) It would be better to include flaA2 in Figure 12, because the control of flaA2 expression is the goal of the presented signaling network.*

(Figure 12) We have included *fla2* in Figure 12 as suggested.

**Reviewer #2**

*Specific comments:*

*1. The authors mention that four bEBPs with a DNA-binding domain have been identified in the C. sphaeroides genome (lines 489-490). This seems to imply that there are bEBPs in C. sphaeroides that lack a DNA-binding domain. Is this correct? If it is true, it would be informative to briefly discuss these bEBPs.*

1. *C*. *sphaeroides* has five EBPs with the conserved motif GA(F/S)TGA which is necessary to interact with RpoN. The EBP FleT lacks the DNA-binding domain, and its transcriptional action is exerted through FleQ, another EBP. As suggested by the reviewer, we have included information about FleT in the Introduction section (lines 79-84). We believe that this information fits well in this section and clarifies the role of this protein.

*2. Was expression of xrpA examined under nitrogen-fixing or limiting nitrogen conditions? The authors report that under heterotrophic growth conditions, xrpA expression was higher when succinate was included as the carbon source versus casamino acids. When using casamino acids as a carbon source, the bacteria presumably are excreting ammonia since the demand of carbon would be greater than the demand for nitrogen. This might explain the lower expression of xrpA with casamino acids as the carbon source.*

2. The expression of *xrpA* has not been examined under nitrogen-fixing or nitrogen-limiting conditions, and the reviewer's suggestion is plausible in explaining the effect of casamino acids. We explored this matter by using the usual amount of (NH_4_)_2_SO_4_ in the culture medium, which corresponds to 3.7 mM, and reducing it to 1.85 mM, 0.74 mM, and 0.37 mM. Total cell extracts of BV11 grown under these conditions did not reveal any notable differences in XrpA levels between the samples. This figure has been uploaded for review.

*3. Bacteria that have two flagellar systems typically elaborate a polar flagellum that is used for swimming and multiple lateral flagella that are used for swarming. That is not the case here, and instead both sets of flagella are used for swimming. It would be helpful if the authors pointed out this distinction.*

3. We have included this information as suggested (lines 93-95).
